# Internet Healthcare Policy Analysis, Evaluation, and Improvement Path: Multidimensional Perspectives

**DOI:** 10.3390/healthcare11131905

**Published:** 2023-06-30

**Authors:** Qi Wei, Xiaoyu Wang, Gongrang Zhang, Xingguo Li, Xuejie Yang, Dongxiao Gu

**Affiliations:** 1 School of Management, Hefei University of Technology, Hefei 230009, China; 2021110918@mail.hfut.edu.cn (Q.W.); grzhang118@126.com (G.Z.); lixingguo@hfut.edu.cn (X.L.); xuejie_y@mail.hfut.edu.cn (X.Y.); 2 The Department of Pharmacy, Anhui University of Traditional Chinese Medicine, Hefei 230009, China

**Keywords:** internet healthcare, policy text analysis, policy instruments, policy evaluation

## Abstract

Internet healthcare is a crucial component of the healthcare industry’s digital transformation and plays a vital role in achieving China’s Healthy China strategy and promoting universal health. To ensure the development of internet healthcare is guided by scientifically sound policies, this study analyzes and assesses current policy texts, aiming to identify potential issues and inadequacies. By examining 134 national-level policy documents, utilizing multiple research methods, including policy bibliometrics, content analysis, and the PMC Index Model, the study investigates policy characteristics, distribution of policy instruments, and evaluation outcomes related to internet healthcare. The study findings reveal that internet healthcare policies place emphasis on enhancing service quality, driving technological innovation, and promoting management standardization. Although policy instruments align with the current stage of internet healthcare development in China, they are plagued by imbalances in implementation. While policies are generally well-formulated, there are discernible discrepancies among them, necessitating the reinforcement and refinement of certain provisions. Hence, it is imperative to strategically optimize the amalgamation and implementation of policy instruments while concurrently endeavoring to achieve a dynamic equilibrium in policy combinations. Furthermore, policymakers should diligently refine the policy content pertaining to its nature and effectiveness in order to fully maximize policy utility.

## 1. Introduction

Internet healthcare refers to a new medical service model that deeply integrates the internet as a carrier and technological means with the healthcare industry, allowing for a more efficient and convenient provision of medical services and health management [1]. With the rapid development of online platforms and digital technologies, Internet healthcare has emerged as a promising avenue to enhance healthcare accessibility, efficiency, and public health outcomes [2,3].

In response to this trend, since 2014, the Chinese government has introduced a range of policy documents, including implementation opinions, development plans, regulatory constraints, and medical insurance payment policies, to support and guide the growth of the internet-based healthcare industry. Based on the characteristics of China’s internet healthcare development and practice, as well as the trend of policies introduced in different years, the development process of internet healthcare can be roughly divided into three stages.

(1) Initial construction phase (2014–2017): As healthcare demand increased and information technology continued to advance, the government implemented a set of policies during this period, such as the “Opinions on Promoting Telemedicine Services” (2014) and the “Guiding Opinions on Actively Promoting the ‘Internet Plus’ Action” (2015), aimed to stimulate the adoption of “Internet Plus” technologies in order to catalyze a significant transformation and growth within the healthcare sector.
(2) Standardization establishment phase (2018–2019): In 2018, the State Council of China issued the “Opinions on Promoting the Development of ‘Internet Plus Healthcare’,” underscoring the imperative of standardization within the internet-based healthcare sector. The government placed significant emphasis on formulating comprehensive service standards and implementing a robust security system to facilitate the industry’s advancement throughout this phase.
(3) Explosive growth phase (2020-present): The outbreak of the COVID-19 pandemic in early 2020 spurred the demand for internet-based healthcare. In response, the government issued a series of policies, including the “Notice on Strengthening Information Technology Support for the Prevention and Control of Novel Coronavirus Pneumonia Epidemic” (2020). These policies covered various areas, such as the construction of smart hospitals, internet-based healthcare insurance services, and online sales of prescription drugs, and aimed to support and regulate the development of the internet healthcare industry.

In the dynamic and transformative landscape of internet healthcare, it is essential for internet healthcare policies to be designed in alignment with the current state of industry development. This enables real-time adjustments to guide and regulate the industry effectively. Reviewing the design and implementation of current internet healthcare policies in China, what are the overall characteristics of these policies? Have the policy instruments and combinations been appropriate? Is the policy design comprehensive and sound? Policy text serves as an objective record of policy intentions and processes [4]. Analyzing policy text is crucial for tracing and observing policy processes, addressing policy concerns, and ensuring the scientific and rational nature of policies [5].

Therefore, the primary objective of this research is to undertake a comprehensive analysis and evaluation of China’s internet healthcare policies by employing methods such as policy bibliometrics, content analysis, and the PMC Index Model to conduct a systematic and multidimensional textual analysis and evaluation of 134 internet healthcare policies issued at the national level between 2014 and 2022. The analysis aims to extract the policy characteristics, distribution of policy instruments, and evaluation results pertaining to internet healthcare and analyze their characteristics and existing issues across three dimensions. The findings suggest that China’s internet healthcare policies suffer from imbalanced use of policy instruments, weak complementarity, and inadequate policy design. Consequently, targeted recommendations will be formulated to enhance the effectiveness of China’s internet healthcare policies, with the ultimate goal of promoting a robust and well-regulated development of internet healthcare.

The remainder of this paper is organized as follows. Section 2 reviews the related literature. Section 3 introduces policy samples and methodology. Section 4 presents the empirical results. Section 5 interprets the finding and concludes this paper.

## 2. Literature Review

As internet healthcare gradually enters the public’s vision, research on internet healthcare policies has become a hot topic of academic concern. Based on the existing research literature, studies on internet healthcare policies can be divided into three aspects.

The first category of research focuses on policy development systems in the field of internet healthcare. Researchers primarily examine the current state of development in internet healthcare and analyze the evolution of internet healthcare policies [6]. Silva et al. [7] conducted an analysis of the relevant regulations in internet healthcare and provided a description of the historical evolution of Brazilian internet healthcare policies across three periods. Ortega et al. [8] analyzed the specific clauses within internet healthcare policies, identifying the problems and challenges encountered during policy implementation and proposed strategies for adjusting internet healthcare services to better meet patient needs. Ekeland et al. [9] utilized qualitative research methods to discuss approaches for achieving quality improvement and necessary digital transformation in the governance of internet healthcare. Huang et al. [10] conducted an evaluation of health informationization in China, considering factors such as health information infrastructure, information technology application, financial and intellectual investment, health resource allocation, and standard systems. Gu et al. [11] employed bibliometric methods to examine the evolution of electronic health and remote medical care, providing insights into the knowledge structure and research hotspots from a global perspective. These studies contribute to a better understanding of the current trends and challenges that researchers face in the field of internet healthcare.

The second area of focus is evaluating the effectiveness of internet healthcare policies. Internet hospitals, which utilize internet technology to provide medical services, are a crucial component of the internet healthcare field. Therefore, most scholars assess the impact of internet healthcare policies by studying internet hospitals. For example, Xie et al. [12] provided an overview of Chinese internet hospitals and evaluated their medical service capabilities. Han et al. [13] summarized the definition and current status of internet hospitals and analyzed the construction and content of Chinese internet hospitals. Zhi et al. [14] used the case of the Zhejiang University Internet Hospital to analyze the operational mode and functional positioning of internet healthcare services. Ouchani and Gholami et al. [15,16] constructed concrete models of hospital management systems using model-based design methodologies to facilitate the integration and communication of medical resources. Lai et al. [17] examined the impact of policy intervention on the development trends and service innovation of internet hospitals through literature analysis and qualitative interviews. Xu et al. [18] analyzed the situation of Chinese internet hospitals during the COVID-19 pandemic, as well as the medical prescription, drug transportation, and medical insurance services they provided.

The third aspect involves the analysis of factors that influence internet healthcare policies and the exploration of relevant facilitating factors for their implementation. Bansal et al. [19] conducted a study that revealed the significant impact of users’ trust in internet healthcare platforms and concerns about privacy on their willingness to utilize such platforms. These factors, in turn, influence the effectiveness of implementing internet healthcare policies. Avanesova et al. [20] identified the critical roles played by national policies, medical capabilities, and infrastructure construction in facilitating the successful implementation of internet healthcare. Luciano et al. [21] identified policies, cultural aspects, and factors related to safety and privacy as important influencers on the implementation of internet healthcare. Powell et al. [22] examined users’ motivations and the facilitating factors that contribute to the utilization of medical services through internet healthcare platforms, and they found that different health behaviors can arise based on various directions of internet healthcare use and users’ access to online health information [23]. Wang et al. [24] proposed that policymakers should prioritize efforts to improve internet access and usage for individuals residing in rural areas, thus bridging the digital divide between urban and rural residents.

Overall, scholars have a theoretical basis for their research on internet healthcare policies. However, the current research lacks depth and systematic analysis (as shown in Table 1). The existing frameworks for policy analysis are primarily based on a single perspective and level, lacking comprehensive and multidimensional research on the internet healthcare policy system. Therefore, this study aims to construct an analysis framework for internet healthcare policies, encompassing “policy topics—policy instruments—policy evaluation.” This study combines qualitative analysis of policy text content with quantitative evaluation of policy implementation effects, taking a multidimensional perspective. The goal is to provide practical and feasible reference guidelines and standards for the design and improvement of future internet healthcare policies.

## 3. Materials and Methods

### 3.1. Research Materials

**Figure 1:**
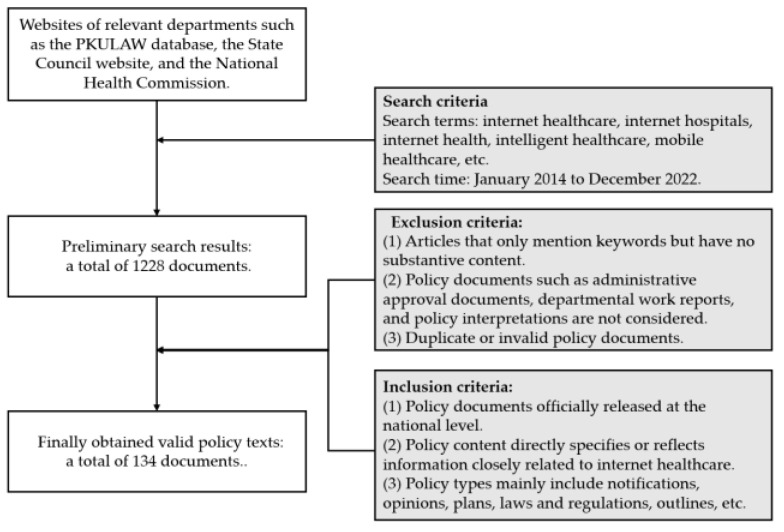
Policy text data collection process.

Considering the hierarchical nature of policy formulation, with local policies being guided by central policies, national policies have a broader influence and representativeness. Furthermore, national policies often embody universal experiences and general principles applicable across different regions. In our analysis, we only selected national-level policy documents on internet healthcare, recognizing their significance in capturing the overarching trends and guiding principles within the field. Multiple data sources were used, including the PKULAW database and websites of relevant departments, such as the State Council and the National Health Commission, with search terms such as “internet healthcare”, “internet hospital”, and “internet health”. Due to the fact that the practical exploration of internet healthcare preceded policy formulation before 2014, the presence of policies during that period was relatively limited. Since 2014, the government has exhibited an increasing level of support and promotion for internet healthcare, resulting in the implementation of a series of significant policies and regulations in the subsequent years, which have generated a sustained impact on the development of internet healthcare. Therefore, the policy retrieval period was limited from 1 January 2014 to 31 December 2022. In order to ensure that policy content is relevant to the theme of internet healthcare, the policy texts were filtered and screened according to inclusion and exclusion criteria, as outlined in Figure 1.

After the screening, a total of 134 valid national policy documents were obtained and numbered in chronological order, as shown in Appendix A.

### 3.2. Research Design

**Figure 2:**
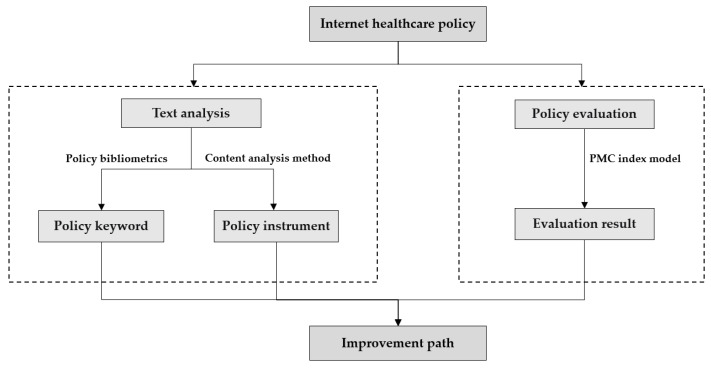
Research framework.

In order to achieve a scientifically rigorous quantitative evaluation, this study situates the policy paradigm within the context of China’s internet healthcare policies. The analysis of policies is broken down into key elements such as policy issues, policy objectives, and policy instruments. A framework of “policy keywords—policy instruments—policy evaluation” is constructed, as outlined in Figure 2. This framework encompasses the comprehensive application of three perspectives.

Firstly, topic keywords were used to represent the content characteristics of policies. Analyzing policies based on topic keywords allows for a reflection of the policy’s core content objectives or issues of the policies, allowing for a better understanding of the overall features and focus of internet healthcare-related policies. It enables a more precise and focused examination of the comprehensive features and focal points of internet healthcare-related policies, thereby enhancing the specificity and targeted nature of the analysis. Thus, this study employed a policy topic keyword perspective and utilized the policy literature’s bibliometrics to conduct word frequency statistics and semantic network analysis on the topic keywords [25], which led to the discovery of the policies’ basic features and points of concern.

Secondly, policy instruments are the tangible manifestation of public policy design and selection, representing the specific means or methods utilized by governments to achieve policy objectives [26]. By analyzing the selection and utilization of policy instruments, it becomes possible to assess the feasibility and effectiveness of policies. This analysis aids in comprehending how policies can purposefully influence the development of internet healthcare. Therefore, this study analyzed the supply, demand, and environmental characteristics of China’s internet healthcare policies using content analysis from the perspective of policy instruments. It dissected the issues of internal balance and matching within policies and interpreted the government’s attention allocation features.

Moreover, policy evaluation represents a crucial component within the process of public policy formulation and management. It involves a comprehensive examination and analysis of policies using scientific evaluation criteria and methods. This process enables not only the scientific assessment of the inherent value of policies but also the examination of the actual effects of policy formulation and implementation [27]. Its significance lies in facilitating the development, execution, and iterative adjustments of internet policies [28]. Hence, this study constructed a PMC index evaluation model from the perspective of policy evaluation to identify deficiencies in policy design across various dimensions and achieve an overall evaluation of the effectiveness of policy implementation.

By integrating the aforementioned three perspectives, a more comprehensive analysis can be conducted to better clarify the core content and objectives of policies, assess the strengths and limitations of policies, and evaluate their feasibility and effectiveness. Lastly, drawing from the systematic multidimensional analysis and aligning with China’s national strategic goals for developing internet healthcare, this study presents targeted recommendations to enhance the effectiveness of internet healthcare policies.

### 3.3. Research Method

#### 3.3.1. Policy Bibliometrics

Policy bibliometrics, as a branch of bibliometrics, is a research method that systematically analyzes and evaluates the structural attributes of policy literature [29]. Its purpose is to uncover the dynamics and trends within the field of policy research by employing quantitative analysis of variables such as the quantity of the policy literature, keywords, authors and institutions, and citation relationships, among others [30]. This approach aims to provide decision support for policy formulation and implementation by shedding light on the evolving landscape of policy studies. Inspired by the principles of bibliometric research pertaining to keywords, this study relied on policy keywords and employed various techniques, including word frequency analysis, co-occurrence analysis, and network analysis, to identify highly specific policy keywords and their interrelationships. Furthermore, it aimed to explore the distinctive characteristics of policies and discern emerging trends within the field.

#### 3.3.2. Policy Instruments

Policy instruments are essential instruments employed by governments to accomplish their policy objectives through a series of measures. Scholars have approached the classification of these instruments from various perspectives. For instance, Howlett et al. [31] categorized policy instruments as voluntary, hybrid, or coercive, depending on the extent of government intervention. On the other hand, McDonnell et al. [32] classified them as incentive-based, command-based, learning-based, or systemic-based, considering the government’s objectives. Rothwell and Zegveld [33] proposed a classification scheme that distinguishes policy instruments as supply-side, environmental, or demand-side based on their impact on technological activities—a widely acknowledged and applied categorization. In this article, we adopted Rothwell and Zegveld’s classification method and organized policy instruments in the domain of internet healthcare into supply-side, demand-side, and environmental types. Moreover, each type is further subdivided into specific policy instruments tailored to the characteristics of internet healthcare, as outlined in Table 2.

#### 3.3.3. PMC Index Model

Presently, widely employed approaches or framework models for policy evaluation, both domestically and internationally, include the Analytic Hierarchy Process (AHP), BP Neural Network Evaluation Method, Fuzzy Comprehensive Evaluation Method, Propensity Score Matching-Difference in Differences (PSM-DID) Method, Grey Relational Model, Dynamic Computable General Equilibrium (CGE) Model, and PMC Index Model, among others. However, many of these methods exhibit certain limitations when applied to policy texts, mainly stemming from inherent subjectivity and relatively lower precision.

The PMC (Policy Modeling Consistency) Index Model, proposed by Ruiz Estrada [34], is a policy evaluation tool that combines traditional text mining methods with advanced mathematical instruments to construct a quantified evaluation model. This model focuses on the raw data of policy texts, aiming to incorporate relevant variables as comprehensively as possible, considering various factors. By assessing the internal consistency of individual policies and enabling comparison among multiple policies, the model effectively embodies the scientific, fair, and rational aspects of the public policy-making process. The analysis process consists of three steps: variable and parameter setting, PMC index calculation, and PMC surface construction.

The first step involves the determination of variables and parameter settings. In this study, reference was made to the research conducted by Ruiz Estrada [35] on policy evaluation, along with relevant explorations [36,37,38,39,40], to identify the criteria for policy evaluation. A total of ten primary variables were selected. Subsequently, taking into consideration the secondary variables discussed by the aforementioned scholars, as well as the analysis of internet healthcare policy text content in Section 4.1 and 4.2, a comprehensive set of 38 secondary variables was established. These variables were based on the extracted policy thematic keywords and policy instruments, allowing for a more nuanced evaluation framework. In the parameter setting, all second-level variables are assigned equal weight, represented in a binary format, as illustrated in Table 3.

The second step involves calculating the PMC (Policy Modeling Consistency) index. Firstly, the binary values of the secondary variables are assigned one by one according to Equation (1). Secondly, the values of each primary variable are calculated according to Equation (2). Finally, the PMC index of each policy is calculated by substituting the values of all primary variables into Equation (3). The policy is then assigned an evaluation level based on Table 4.

(1) $$X∼N\left[0,1\right];X=\mathrm{XR}:\left[0∼1\right]$$

(2) $$X_{t}\left(\overset{n}{\underset{j=1}{\sum }}\;\frac{X_{ij}}{T\left(X_{tj}\right)}\right)t=1,2,3,\cdots ,$$

Among them, t = 1, 2, 3, …, *n*; t is the first-level variable, and *j* is the second-level variable.

(3) $$\begin{array}{cc} \;[X_{1}(\overset{5}{\underset{i=1}{\sum }}\;\frac{X_{1i}}{5})\;+ & X_{2}(\overset{3}{\underset{j=1}{\sum }}\;\frac{X_{2j}}{3})+X_{3}(\overset{4}{\underset{k=1}{\sum }}\;\frac{X_{3k}}{4})+X_{4}(\overset{4}{\underset{l=1}{\sum }}\;\frac{X_{4l}}{4})+X_{5}(\overset{3}{\underset{m=1}{\sum }}\;\frac{X_{5m}}{3})\\ & +X_{6}(\overset{4}{\underset{n=1}{\sum }}\;\frac{X_{6n}}{4})+X_{7}(\overset{5}{\underset{o=1}{\sum }}\;\frac{X_{7o}}{5})+X_{8}(\overset{6}{\underset{p=1}{\sum }}\;\frac{X_{8p}}{6})+X_{9}(\overset{4}{\underset{q=1}{\sum }}\;\frac{X_{9q}}{4})]\; \end{array}$$

The third step involves constructing the PMC surface. The PMC surface presents the evaluation results and advantages and disadvantages of policies in a three-dimensional image, allowing for an intuitive display of the results [41]. In this study, the evaluation samples selected are all publicly available policies, and *X*10 has no effect on the evaluation results. Therefore, *X*10 is removed to ensure the symmetry and balance of the matrix. The policy evaluation third-order matrix is constructed from *X*1–*X*9, and the surface plots of each policy are drawn using the calculation method shown in Equation (4).

(4) $$\mathrm{PMC}\mathrm{surface}=\left[\begin{array}{ccc} X_{1} & X_{2} & X_{3}\\ X_{4} & X_{5} & X_{6}\\ X_{7} & X_{8} & X_{9} \end{array}\right]$$

## 4. Results

### 4.1. Policy Bibliometrics

#### 4.1.1. Analysis of Word Frequency

**Figure 3:**
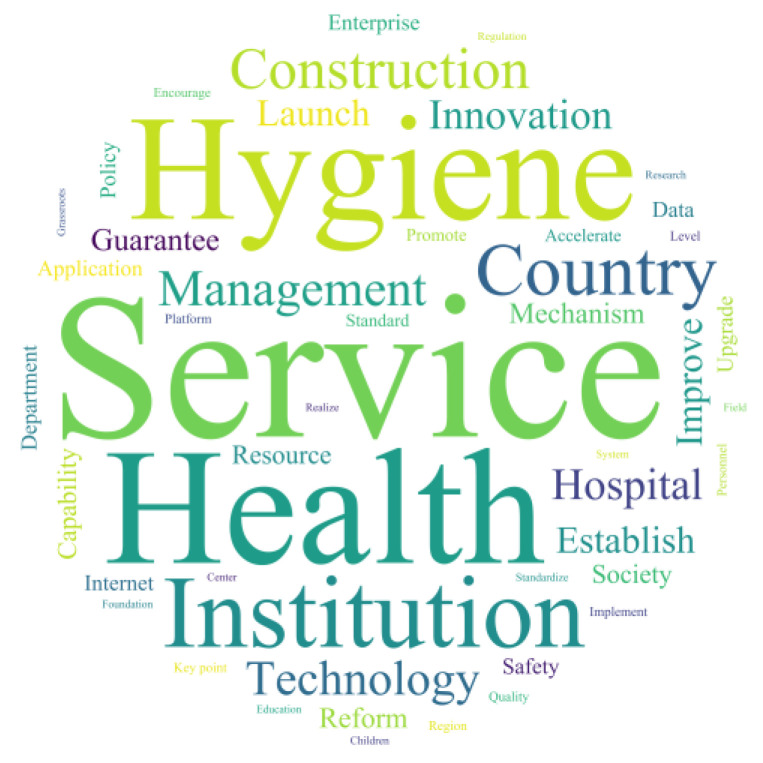
Policy text word cloud.

In this study, the Rost CM6 software was used to perform word segmentation on 134 policy texts with a minimum length of two characters. Interference words were filtered out through manual judgment to remove irrelevant words such as adverbs and mood words. High-frequency words were extracted, and Table 5 shows the top 30 theme words ranked by frequency in the policy texts. Word Cloud was used to visualize the filtered high-frequency words into a word cloud, as shown in Figure 3.

The data shows that the three most frequently appearing words in the policy texts are *“*service*”* (8496 times), *“*medical*”* (7289 times), and *“*health*”* (5120 times), indicating that the main focus of China*’*s internet healthcare is on providing services and promoting health. The themes can be further categorized into four dimensions: entities, technology, management, and services.

Entities include the government, institutions, hospitals, and patients. One of the primary objectives of internet healthcare is to foster communication and collaboration between medical professionals and patients, thereby enhancing the quality and efficiency of healthcare services. The pivotal role played by these stakeholders is instrumental in achieving this objective. With the support of internet platforms, healthcare practitioners and patients can conveniently engage in activities such as medical consultations, appointment scheduling, and telemedicine, facilitating mutual interaction and collaboration between both parties.

Technology includes big data, internet, and artificial intelligence. One of the primary objectives of internet healthcare is to leverage technological advancements to improve the effectiveness of healthcare services and management. The continuous development and innovative applications of technology have facilitated the convenient and efficient acquisition, transmission, and processing of medical information. This enables healthcare professionals to access comprehensive medical knowledge and receive enhanced decision-making support. Simultaneously, patients benefit from more accessible healthcare services and gain access to sophisticated instruments for health management.

Management includes development, innovation, establishment, and improvement. One of the fundamental goals of internet healthcare is to establish a comprehensive management system that encompasses the development of policies and regulations, data security and privacy protection measures, as well as healthcare quality management protocols. Effective management plays a pivotal role in safeguarding the security and reliability of internet healthcare, while also enhancing the standardization and credibility of healthcare services. Moreover, it provides healthcare professionals and patients with a favorable healthcare experience, along with necessary safeguards.

Services include medical services, health, hygiene, diagnosis, and treatment. One of the primary goals of internet healthcare is to facilitate improved healthcare service experiences and enhanced convenience. Internet-based healthcare enables patients to access medical consultations, schedule appointments, and purchase medications at their convenience, regardless of time and location. Moreover, healthcare professionals can engage in activities such as remote diagnosis and tele-surgical guidance. These advancements significantly streamline medical communication and service provision between patients and healthcare providers, fostering greater accessibility and convenience in the healthcare delivery process.

In conclusion, the interconnected dimensions of stakeholders, technology, management, and services synergistically contribute to the attainment of the goals set forth in internet healthcare. These dimensions collectively propel the development and innovation of internet healthcare, facilitating the provision of convenient, efficient, and high-quality healthcare services. By strategically integrating and harmonizing these dimensions, the healthy advancement of internet healthcare can be fostered, resulting in heightened healthcare service quality and efficiency, as well as an improved healthcare experience and overall well-being for patients.

#### 4.1.2. Analysis of the Semantic Network

**Figure 4:**
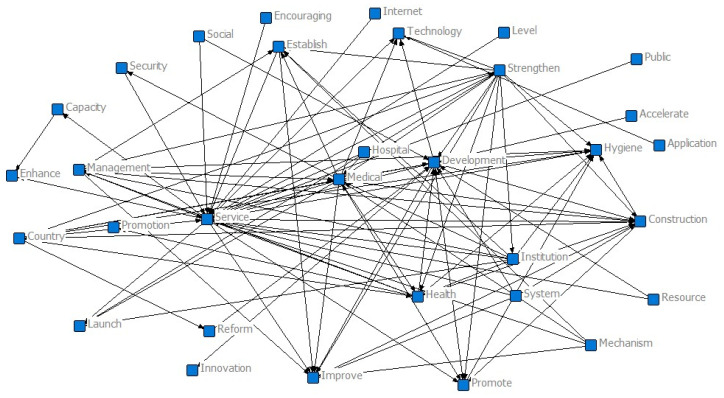
Semantic network of the internet healthcare policy.

Furthermore, we conducted a co-occurrence analysis on the theme words to generate a co-occurrence matrix, revealing the degree of association between them, as shown in Table 6. A higher frequency of co-occurrence indicates a stronger correlation between the themes. We also utilized the net draw instrument to generate a semantic network graph, which provides a visual representation of the core content and connections of the policy text, as depicted in Figure 4.

From Figure 4, it can be seen that *“*Service*”* is at the core position and is a hot topic in Internet healthcare policies. It is closely related to key topics such as *“*Health*”*, *“*Development*”*, *“*Management*”*, *“*Strengthening*”*, and *“*Technology*”*. This indicates that the policy content mainly focuses on the service nature of internet healthcare services, emphasizes the innovation and application of medical technology, and strengthens and improves medical management. *“*Medical*”* is the foundation guarantee of internet healthcare policies, with related keywords such as *“*Institutions*”*, *“*Construction*”*, *“*Hospitals*”*, and *“*Guarantee*”*, reflecting that policy content revolves around the construction of medical institutions and medical guarantees. *“*Healthcare*”* is the direction emphasized in internet healthcare policies, with related words such as *“*Innovation*”*, *“*Improvement*”*, *“*Resources*”*, *“*Reform*”*, and *“*System*”*, reflecting that the policy*’*s main focus is on improving the healthcare system, innovation, and allocation of related resources. These keywords form a systematic focus of the policies, showing the content and structure of Internet healthcare policy texts as a whole.

### 4.2. Policy Instrument

Based on the classification of policy instruments mentioned above, specific policy items were taken as the unit of content analysis to code the text of internet healthcare policies, with the content analysis process carried out using the MAXQDA qualitative data analysis software. After the initial coding, multiple revisions were made to eliminate controversial coding terms. Eventually, 739 policy instrument coding units were obtained, and the classification results are presented in Table 7.

(1) Bias in policy supply and demand

Overall, China*’*s internet healthcare policies consider the use of supply-side, demand-side, and environmental policy instruments. However, there are significant differences in the extent to which these three policy instruments are utilized. Supply-side policy instruments are the most frequently used, with 362 measures accounting for 50.0% of the total and playing a leading role. Environmental policy instruments, with 304 measures accounting for 41.1%, are also commonly used. On the other hand, demand-side policy instruments are used less frequently, with only 73 measures, accounting for 9.9%, and they are relatively scattered. This overall reflects a *“*top-down*”* supply-side policy orientation dominated by the government, but the limited pull effect of demand-side policy instruments results in a significant deviation between supply and demand, which in turn affects the overall effectiveness of the development of internet healthcare.

(2) Uneven use of policy instruments

In practical application, there are differences in the use of the three types of policy instruments. Within the supply-side policy instruments, the government places a greater emphasis on information technology support (31.2%), public services (30.7%), and infrastructure construction (23.2%). This is because information technology is the fundamental support for the construction of the internet healthcare service system and plays an important role in improving the efficiency and quality of medical services. At the same time, the government also actively uses internet technology and platforms to provide medical and health services to the general public and vigorously strengthens the construction of infrastructure to ensure the interconnection of information and remove obstacles to the development of internet healthcare services. However, the application of talent cultivation (4.1%) and funding (1.4%) instruments is relatively low. Nevertheless, funding and talent are key factors in promoting the construction of the internet healthcare service system, and the absence of these two factors will affect the effectiveness of medical services, thereby restricting the development of Internet healthcare and health.

In terms of environmental policy instruments, the government has a greater focus on technical standards (46.4%) and legal regulation (32.9%). This reflects the fact that as a new concept, internet healthcare needs to be developed based on corresponding technical standards. Meanwhile, the government needs to adopt regulatory control to regulate and effectively supervise the behavior of various entities participating in internet healthcare services, ensuring the steady development of internet healthcare. The use of target planning instruments (11.8%) is the second most frequent, but the application of organizational coordination (5.6%) and publicity and promotion (3.3%) instruments is relatively low and neglected. To ensure the smooth promotion of internet healthcare, the government should strengthen the use of these two categories of demand policy instruments to create a favorable environment for the development of internet healthcare.

Among the demand-driven policy instruments, pilot standardization (42.5%) and medical insurance payment (30.1%) have received the most attention, indicating that the government attaches great importance to legislative pilot precedents and has gradually included internet healthcare services in the scope of medical insurance to drive the development of internet medicine. Public–private partnerships (23.3%) come next, but there is a significant gap in the use of international exchange instruments (4.1%). However, as internet healthcare has become an important global trend, international exchange and cooperation are crucial to promoting its development and progress. In the future, it is necessary to strengthen cooperation between countries and jointly promote the globalization of internet healthcare.

(3) Use of policy instruments in line with development history

In light of the stages of development in internet healthcare, as demonstrated in Table 8, there are notable distinctions in the utilization and allocation of policy instruments across various phases.

The usage of supply-side policy instruments is primarily concentrated during the initial construction phase, accounting for 67% of policy instrument utilization. This indicates that the government intends to increase resource inputs, such as manpower, materials, and finance, to promote the development of internet healthcare. Environmental policy instruments are mainly used during the standardization establishment phase and the explosive growth phase, with a utilization rate of 49% during both stages. The aim is to address legal and technical standard gaps, support internet healthcare in responding to development opportunities and risks, create a favorable policy environment, and indirectly promote its development. The utilization of standard and regulatory instruments increases for each phase, promoting the standardization and rationalization of internet healthcare services. The utilization of demand-driven policy instruments has a small variation in percentage across the three stages, accounting for 10%, 12%, and 8% during the initial construction phase, standardization establishment phase, and explosive growth phase, respectively. The utilization of medical insurance payment instruments increases during each phase, promoting the sustainable development of internet healthcare services. It is evident that the government has made a rational selection of policy instruments to address the development requirements and policy demands of different phases when formulating internet healthcare policies.

### 4.3. PMC Index Evaluation

#### 4.3.1. Selection of the Sample

The PMC Index Model is designed for analyzing and studying specialized policies, while policies related to internet healthcare are mostly comprehensive, with provisions dispersed across comprehensive documents such as *“*Internet Plus*”*, deepening medical and health system reform, and the 13th and 14th Five-Year Plans, among others. This study further screened the collected policy documents and selected 16 specialized representative policies as evaluation samples to ensure the reasonableness and comprehensiveness of the policy sample, as shown in Appendix B.

#### 4.3.2. PMC Index Calculation and Surface Construction

**Figure 5:**
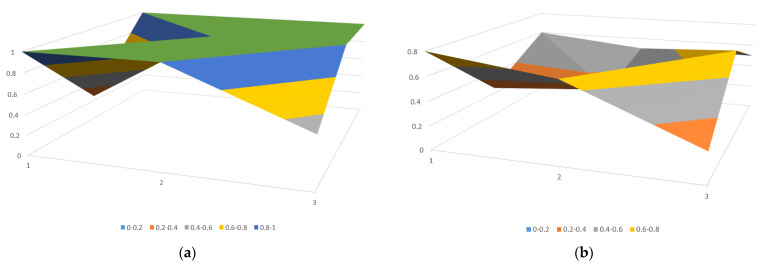
(**a**) PMC surface of P4, and (**b**) PMC surface of P2.

According to the previously established steps, the PMC index scores for each policy were calculated, and the scores were sorted and classified into levels, as shown in Table 9. Surface plots were generated through three-dimensional visualization to demonstrate the evaluation scores and strengths and weaknesses of policies. The presence of surface undulations and color variations indicates differences in policy scores. Raised areas correspond to higher policy evaluation scores, while recessed areas correspond to lower policy evaluation scores. This study presents the PMC surfaces of the policy with the highest PMC index score (P4) and the lowest score (P2), as shown in Figure 5.

#### 4.3.3. Analysis of Evaluation Results

(1) Longitudinal evaluation

According to the PMC index results in Table 9, it can be seen that all policies have a membership level of either excellent or qualified. The mean PMC index of the 16 policies is 7.31. The order of policy scores from high to low is P4 > P1 > P8 > P16 > P3 > P5 > P6 > P14 > P7 > P11 > P15 > P13 > P12 > P10 > P9 > P2.

Grade A excellent policies are policies with a score between 7 to 8.99 points and a policy grade of excellent. They include P1, P3, P4, P5, P6, P7, P8, P11, P14, and P16. For instance, P4 has a PMC index of 8.75; this policy is an opinion on the development of health and medical big data application issued by the State Council, ranking first. The PMC surface plot depicted in Figure 5a showcases a predominance of convex surfaces, indicating that P4 scored higher across a range of indicators. In terms of specific scores, 8 out of 10 primary variables received the highest score, indicating that the policy*’*s overall design is scientifically reasonable and its content covers all indicators in the evaluation model. To further enhance this policy, optimization of the policy level (*X*4) increased cross-departmental collaboration and supervision and ensured the policy*’*s effectiveness and implementation could be considered to promote the development of medical big data.

Grade B qualified policies are those with a score ranging from 5 to 6.99 points and a policy grade of qualified. They include P2, P9, P10, P12, P13, and P15. For example, P2 has a PMC index of 5.65. This policy is a set of opinions issued by the National Health and Family Planning Commission regarding the promotion of telemedicine services in healthcare institutions, ranking sixteenth. The PMC surface plot depicted in Figure 5b shows fewer convex points on the surface plot, and most of the surfaces are concave, indicating that P2 received lower scores across various indicators, reflecting its relative deficiencies compared to other policies. In terms of specific scores, 9 out of 10 primary variables require further improvement, with the policy objectives (*X*5) score significantly lower than the average. Therefore, the scope of policy recipients needs to be expanded further. Furthermore, the scores for policy instruments (*X*2), policy goals (*X*6), policy functions (*X*7), incentives and constraints (*X*8), and policy evaluation (*X*9) are also low. This indicates that the policy*’*s mode of action is relatively single, and its coverage is relatively narrow. It is recommended to implement a multi-stage and well-planned approach to address these concerns. A more scientific and effective improvement path can be pursued by considering the difference between variable scores and the mean value. The suggested path for improvement is as follows: *X*5→*X*9→*X*8→*X*7→*X*2→*X*6→*X*4→*X*1→*X*3.

(2) Cross-sectional evaluation

**Figure 6:**
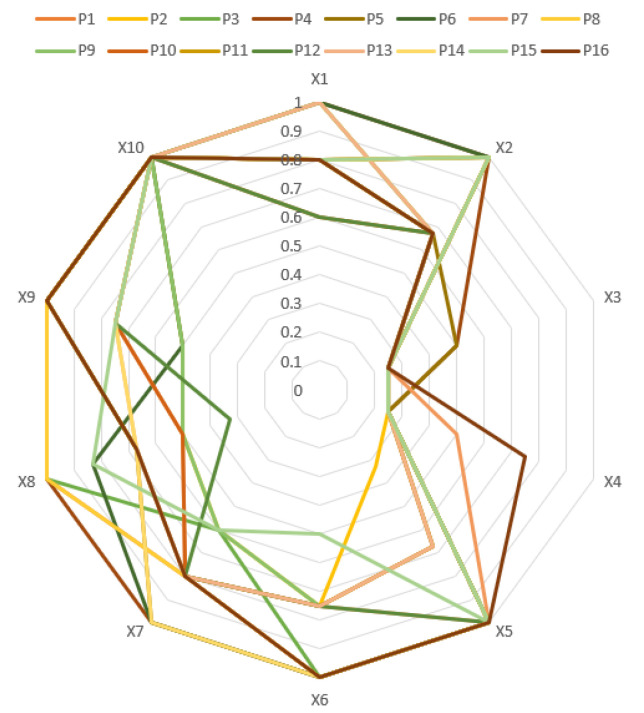
Radar chart of first-level indicator scores.

This paper generated a radar chart based on the scores of their primary indicators to facilitate the comparison and evaluation of the selected 16 policies, as illustrated in Figure 6.

The results indicate that overall, the policies performed well, but there are still some shortcomings in individual indicators. Looking at the average scores of each indicator, *X*1, *X*2, *X*5, *X*6, and *X*10 have relatively high average scores, while *X*3 and *X*4 have the lowest average scores. Specifically, the average score for policy nature (*X*1) is 0.84, indicating that most policies focused on description, suggestion, guidance, and supervision but had less involvement from a predictive perspective. The average score for policy instruments (*X*2) is 0.84, with supply-side and environmental policy instruments being widely used in most policies but demand-side policy instruments being less frequently used. The average score for policy effectiveness (*X*3) is 0.28, indicating that most policies had only short-term effects, suggesting that the development target deadline for internet healthcare policies is relatively narrow. The average score for policy level (*X*4) is 0.3, indicating that most policies are issued by single-type entities, and cooperation between different levels of departments needs to be strengthened. The average score for policy objects (*X*5) is 0.87, with all policies involving the participation of medical institutions and government agencies, but some policies lack cooperation with third-party institutions. The average score for policy goals (*X*6) is 0.86, with all policies promoting health and implementing corresponding measures to optimize services, but some policies lack measures for standard setting and resource balance. The average score for policy functions (*X*7) is 0.8, with all policies including functions for service optimization, supervision and constraints, and normative guidance, but some policies lack functions for innovation-driven and industry support. The average score for incentive constraints (*X*8) is 0.71, with areas for improvement in talent development and funding, especially in the lack of specific measures for funding. The average score for policy evaluation (*X*9) is 0.81, with all policies based on sufficient evidence and scientific programs but with relatively thin descriptions of objectives and content, needing to strengthen this aspect of expression. The average score for policy openness (*X*10) is 1, indicating that all policies were open policies.

## 5. Conclusions

This study has established a framework of “policy keywords—policy instruments—policy evaluation” to analyze and evaluate the internet healthcare policies in China from a multidimensional perspective, as shown in Table 10. The research findings demonstrate that the content of internet healthcare policies primarily revolves around the aspects of user interaction, service quality, technological innovation, and regulatory standards, aiming to promote the development of internet healthcare and enhance its efficiency and quality. However, the usage of policy instruments in internet healthcare policies generally aligns with the overall development trajectory of internet healthcare in China but also exhibits an imbalanced pattern and lacks complementarity. Among the policy instruments used, there is a greater emphasis on supply-side and environmental instruments, while the attention given to demand-side instruments is insufficient, thus slowing down the progress of “Internet healthcare.” Additionally, there are significant gaps in the supply of instruments related to funding investment and talent development, deficiencies in environmental aspects such as promotion and organizational coordination, and a lack of international exchange and cooperation on the demand side. The analysis using the PMC Index Model indicates an average PMC index of 7.14, reflecting a generally favorable situation of internet healthcare policies. However, there exist variations in the effectiveness of different policies, and some policy provisions require improvement in terms of efficacy, objectives, and functions, highlighting the need for strengthening and enhancing the policies.

Furthermore, it is recognized that internet healthcare policies worldwide encounter common challenges, such as imbalanced use of policy instruments, weak complementarity among policies, and inadequate policy design due to rapid technological advancements. These challenges impede the effective regulation of online healthcare services.

Based on the aforementioned analysis, the following policy recommendations are proposed:

(1) Achieving a balanced utilization of policy instruments is crucial. Particularly, it is essential to enhance the combination and application of demand-side policy instruments, as they directly stimulate policy actors and yield significant effects [42]. Considering the constraints posed by limited public fiscal revenue in China, policies should be employed as the primary means to propel the development of internet healthcare. This can be achieved by attracting increased participation from various social forces in areas and segments aligned with intelligent healthcare and online medical services, thereby alleviating the policy’s financial, technological, and human resource burdens [43]. Furthermore, policy formulation should emphasize the importance of knowledge exchange and cooperation among different nations in the realm of internet healthcare, facilitating resource integration and sharing to collectively foster the advancement of internet healthcare endeavors;
(2) Strengthen talent cultivation and fiscal investment. In the context of future policy formulation, it is crucial to reinforce the mechanisms for talent cultivation and professional development in the realm of internet healthcare. Particular emphasis should be placed on nurturing highly skilled individuals capable of effectively integrating knowledge in internet technologies and medical domains. Furthermore, attention ought to be directed toward augmenting governmental fiscal investment in the establishment of internet healthcare service systems. Encouragement and support should be extended to healthcare institutions at various levels, empowering them to leverage pertinent information technologies, notably the internet, to deliver healthcare services, thus fostering enhancements in service quality and capacity. The government is urged to intensify its efforts in amplifying the outreach and promotion of policies to ensure the smooth progress of internet healthcare [44]. This necessitates the broad utilization of diverse communication channels to enhance the dissemination and interpretation of internet healthcare policies. Additionally, a coordinated approach involving pertinent departments must be fostered, engendering collaborative mechanisms that engender an enabling environment for the development of internet healthcare;
(3) Undertake an integrated and systemic evaluation of internet healthcare policies. In the realm of policy formulation, it is imperative to establish a robust mechanism for conducting comprehensive and systemic evaluations of internet healthcare policies. Due emphasis should be placed on developing an evaluative framework that encompasses the holistic nature of policies moving forward, ensuring a thorough assessment of their efficacy, objectives, and functionalities. This approach safeguards the integrity, scientific rigor, and feasibility of the policies. At present, as policies in the domain of internet healthcare undergo refinement, it is crucial to accord significant importance to long-term planning, laying a solid foundation for sustainable growth. Furthermore, policy improvement necessitates expanded coverage that enhances the functionality of the policies. Within the ambit of long-term planning, it becomes indispensable to align strategic objectives with attainable medium- and short-term targets, supplemented by periodic evaluations of policy outcomes at different stages.

Lastly, due to the predominantly macroscopic perspective adopted during policy formulation, certain provisions within the policies may possess a high degree of generality, lacking specific guiding principles. As a result, the practical effectiveness of policy implementation may be suboptimal. Future policy implementation endeavors should entail further elaboration of execution plans by policymakers to address this. This refinement process should prioritize ensuring the policy’s comprehensibility and operational feasibility. Concurrently, policy executors should diligently formulate corresponding implementation schemes and strategies that align with specific requirements and guidelines. This approach serves to facilitate the seamless execution and effective implementation of policies. Furthermore, augmenting oversight and evaluation mechanisms throughout the policy execution process is imperative [45]. This proactive approach allows for the timely identification of challenges and areas requiring improvement, thereby augmenting the efficacy of policy implementation.

## Figures and Tables

**Table 1:** Summary of the literature on internet healthcare policies research.

Research Topic	Related Work	Pros	Cons
Study of the policy development system	Reviewing the current status and evolution of internet healthcare policies	Uncover the background, environment, and significance inherent in policy changes and explore the development and changes in internet healthcare	Limited research perspective, relying on superficial qualitative descriptive analysis
Evaluation of the implementation effectiveness of internet healthcare policies	Studying internet hospitals and evaluating the implementation effectiveness of internet healthcare policies	Examine the scientific and feasibility of internet healthcare policies in light of the actual development of internet healthcare	Inadequate comprehensiveness of research content and excessive subjectivity in evaluation
Analysis of factors influencing the implementation of internet healthcare policies	Using empirical analysis to explore the factors influencing the implementation of internet healthcare policies	Enhanced understanding and better response to various factors involved in policy formulation and implementation	More focus on the main subjects of internet healthcare policies and some external environmental factors, insufficient emphasis on policy content

**Table 2:** Classification and meaning of internet healthcare policy instrument.

Instrument Type	Specific Name	Meaning
Supply	Infrastructure construction	Regulate the relevant infrastructure construction for the development and application of internet healthcare.
	Talent cultivation	Various education and training activities for practitioners of internet healthcare strengthen the training of relevant talents.
	Fund investment	Provide financial and technological support, including setting up special funds and subsidies, etc.
	Public services	Promote the integration of the internet and medical healthcare services, improving the internet healthcare service system.
	Information technology support	Build relevant databases and knowledge bases, utilizing modern information technology to provide information exchange and medical services.
	Resource allocation	Government macro-control of resources, coordinating resource allocation, and promoting equalization of basic public health.
Environment	Target planning	Establish macro targets and overall planning, determining the development direction of internet healthcare.
	Legal regulation	Normative and regulatory measures on the behavior of medical service providers through laws and systems ensure orderly development.
	Technical standards	Construct the relevant technical standards and evaluation system of internet healthcare services.
	Promotion	Active promotion of the policy measures and progress of internet healthcare, generating positive public opinion.
	Organizational coordination	The government establishes a special leadership organization to coordinate the management of internet healthcare services and promote overall planning.
Demand	Pilot standardization	Conduct pilot projects and standardization of internet healthcare service projects.
	Medical insurance payment	Promote the reform of internet diagnosis, treatment, and payment models, expanding the medical insurance coverage of internet healthcare.
	International exchanges	Exchange knowledge and services of internet healthcare with other countries to learn from advanced foreign experiences.
	Public–private partnerships	The government encourages and guides social capital to participate in cooperation.

**Table 3:** Evaluation index and standard setting for internet healthcare policies.

First-Level Variables	Second-Level Variables	Evaluation Standards
X1 Policy nature	X1:1 Predictive X1:2 Advisory X1:3 Descriptive X1:4 Guiding X1:5 Regulatory	Judge whether the policy content has predictive, advisory, descriptive, guiding, and regulatory characteristics. If yes, the score is 1; otherwise, the score is 0.
X2 Policy instruments	X2:1 Supply-side X2:2 Demand-side X2:3 Environment-side	Judge whether the policy instruments are supply-side, demand-side, or environment-side. If yes, the score is 1; otherwise, the score is 0.
X3 Policy effectiveness [36]	X3:1 Temporary X3:2 Short-term X3:3 Medium-term X3:4 Long-term	Judge whether the policy impact is within one year, 1–5 years, 6–10 years, or more than 10 years. If yes, the score is 1; otherwise, the score is 0.
X4 Policy level [37]	X4:1 State Council X4:2 State Council departments X4:3 State Council-affiliated institutions X4:4 National bureaus managed by departments	Judge whether the policy issuer is the State Council, State Council departments, State Council-affiliated institutions, or national bureaus managed by departments. If yes, the score is 1; otherwise, the score is 0.
X5 Policy objectives	X5:1 Government institutions X5:2 Medical institutions X5:3 Third-party institutions	Judge whether the policy objectives include government institutions, medical institutions, or third-party institutions. If yes, the score is 1; otherwise, the score is 0.
X6 Policy goals	X6:1 Health X6:2 Services X6:3 Resources X6:4 Standards	Judge whether the policy goals are to promote health, optimize services, balance resources, or establish standards. If yes, the score is 1; otherwise, the score is 0.
X7 Policy functions [38]	X7:1 Regulatory constraints X7:2 Normative guidance X7:3 Service optimization X7:4 Innovation-driven X7:5 Industrial support	Judge whether the policy has functions of regulatory constraints, normative guidance, service optimization, innovation-driven, or industrial support. If yes, the score is 1; otherwise, the score is 0.
X8 Incentives and constraints [39]	X8:1 Legal protection X8:2 Supervision and assessment X8:3 Skill training X8:4 Talent introduction X8:5 Capital investment X8:6 Organizational implementation	Judge whether incentives and constraints involve legal protection, supervision, assessment, skill training, talent introduction, capital investment, or organizational implementation. If yes, the score is 1; otherwise, the score is 0.
X9 Policy evaluation [40]	X9:1 Sufficient evidence X9:2 Clear objectives X9:3 Scientific plan X9:4 Detailed content	Judge whether the policy evaluation is based on sufficient evidence, and has clear objectives, a scientific plan, and detailed content. If yes, the score is 1; otherwise, the score is 0.
X10 Policy transparency	—	Judge whether the policy is transparent. If yes, the score is 1; otherwise, the score is 0.

**Table 4:** Policy PMC index evaluation level.

PMC Score	9–10	7–8.99	5–6.99	0–4.99
Evaluation Level	Optimal	Excellent	Qualified	Not Up to Standard

**Table 5:** High-frequency policy themes (excerpt).

Keyword	Frequency	Keyword	Frequency
Service	8496	Launch	2112
Medical	7289	Improve	2069
Health	5120	Guarantee	2010
Development	4687	Mechanism	1864
Hygiene	4603	Reform	1838
Institution	3931	Resource	1816
Country	3383	Society	1783
Construction	3312	Promote	1750
Strengthen	3301	Capability	1707
Management	3270	System	1706
Technology	2873	Application	1691
Promote	2351	Department	1601
Hospital	2336	Safety	1585
Innovation	2316	Internet	1562
Establish	2179	Enterprise	1561

**Table 6:** Co-occurrence matrix (excerpt).

Keyword	Service	Medical	Health	Development	Hygiene	Institution	Country	Construction	Strengthen	Management
Service	8496	1878	1188	1111	1289	1273	681	990	1027	1078
Medical	1878	7289	1169	756	1491	1610	590	704	882	947
Health	1188	1169	5120	833	1448	690	805	666	724	712
Development	1111	756	833	4687	691	0	898	811	722	0
Healthcare	1289	1491	1448	691	4603	1068	920	648	743	703
Institution	1273	1610	690	0	1068	3931	0	0	676	704
Country	681	590	805	898	920	0	3383	587	558	0
Construction	990	704	666	811	648	0	587	3312	973	632
Strengthen	1027	882	724	722	743	676	558	973	3301	800
Management	1078	947	712	0	703	704	0	632	800	3270

**Table 7:** Distribution of various policy instruments.

Instrument Type	Proportion	Specific Instrument	Frequency	Proportion
Supply (326)	50%	Information technology support	113	31.2%
		Public services	111	30.7%
		Infrastructure construction	84	23.2%
		Resource allocation	34	9.4%
		Talent cultivation	15	4.1%
		Fund investment	5	1.4%
Environment (304)	41.1%	Technical standards	141	46.4%
		Legal regulation	100	32.9%
		Target planning	36	11.8%
		Organizational coordination	17	5.6%
		Promotion	10	3.3%
Demand (73)	9.9%	Pilot standardization	31	42.5%
		Medical insurance payment	22	30.1%
		Public-private partnerships	17	23.3%
		International exchanges	3	4.1%

**Table 8:** Distribution of policy instruments in different development stages.

Instrument Type	Specific Instrument	2014–2017	2018–2019	2020–2022
Supply	Infrastructure construction	29	26	27
	Resource allocation	18	8	8
	Public services	39	35	37
	Information technology support	58	15	42
	Fund investment	2	2	1
	Talent cultivation	4	7	4
Environment	Technical standards	22	39	80
	Legal regulation	10	56	34
	Target planning	14	16	6
	Organizational coordination	5	4	8
	Promotion	1	3	6
Demand	Medical insurance payment	2	9	11
	Pilot standardization	9	13	9
	International exchanges	2	0	1
	Public–private partnerships	9	6	2

**Table 9:** PMC index of 16 internet healthcare policies.

	*X*1	*X*2	*X*3	*X*4	*X*5	*X*6	*X*7	*X*8	*X*9	*X*10	PMC Index	Rating	Ranking
P1	0.8	1	0.25	0.25	1	1	0.8	1	1	1	8.1	Excellent	2
P2	0.8	0.67	0.25	0.25	0.33	0.75	0.6	0.5	0.5	1	5.65	Qualified	16
P3	0.8	1	0.25	0.25	1	1	0.6	1	1	1	7.9	Excellent	5
P4	1	1	0.5	0.25	1	1	1	1	1	1	8.75	Excellent	1
P5	0.8	0.67	0.5	0.25	1	1	1	0.67	1	1	7.89	Excellent	6
P6	1	1	0.25	0.25	1	1	1	0.83	0.5	1	7.83	Excellent	7
P7	0.8	1	0.25	0.5	1	0.75	0.8	0.5	0.75	1	7.35	Excellent	9
P8	1	0.67	0.25	0.25	1	1	0.8	1	1	1	7.97	Excellent	3
P9	1	0.67	0.25	0.25	0.67	0.75	0.6	0.5	0.5	1	6.19	Qualified	15
P10	0.6	0.67	0.25	0.25	0.67	0.75	0.8	0.5	0.75	1	6.24	Qualified	14
P11	0.8	1	0.25	0.25	0.67	0.75	0.8	0.67	1	1	7.19	Excellent	10
P12	0.6	0.67	0.25	0.25	1	0.75	0.8	0.33	0.75	1	6.4	Qualified	13
P13	1	0.67	0.25	0.25	0.67	0.75	0.8	0.67	0.75	1	6.81	Qualified	12
P14	0.8	1	0.25	0.25	1	1	1	0.67	0.75	1	7.72	Excellent	8
P15	0.8	1	0.25	0.25	1	0.5	0.6	0.83	0.75	1	6.98	Qualified	11
P16	0.8	0.67	0.25	0.75	1	1	0.8	0.67	1	1	7.94	Excellent	4
Average	0.84	0.84	0.28	0.30	0.88	0.86	0.8	0.71	0.81	1	7.30		

**Table 10:** Summary of research findings.

Research Perspective	Research Work	Finding
Policy keywords	This study utilized the policy literature’s bibliometrics to conduct word frequency statistics and semantic network analysis on the topic keywords.	The content of internet healthcare policies primarily revolves around the aspects of user interaction, service quality, technological innovation, and regulatory standards.
Policy instruments	This study employed content analysis to analyze the supply, demand, and environmental characteristics of policies.	The usage of policy instruments generally aligns with the overall development trajectory of Internet healthcare but also exhibits an imbalanced pattern and lacks complementarity.
Policy evaluation	This study constructed a PMC index evaluation model to identify deficiencies in policy design across various dimensions	The policies are generally well-formulated, but there are still some shortcomings in individual indicators.

## Data Availability

The data used to support the findings of this study are available from the corresponding author upon request.

## Appendix A

**Table A1:** China’s internet healthcare policy document for 2014–2022.

NO.	Policy Document	Issuing Department	Issuing Date
P1	Notice on the issuance of key tasks for deepening the reform of the medical and health system in 2014	Office Council of The State	13 May 2014
P2	Notice on the issuance of the measures for the management of village health offices (for trial implementation)	National Health and Family Planning Commission, National Development and Reform Commission, Ministry of Education, etc.	3 June 2014
P3	Notice on telemedicine policy for elderly institutions	National Development and Reform Commission, Ministry of Civil Affairs, National Health and Family Planning Commission	16 June 2014
P4	Opinions on promoting telemedicine services in medical institutions	National Health and Family Planning Commission	21 August 2014
P5	Opinions on promoting the healthy development of smart cities	National Development and Reform Commission, Ministry of Industry and Information Technology, Ministry of Science and Technology and other eight departments	27 August 2014
P6	Opinions on accelerating the development of commercial health insurance	Office Council of The State	27 October 2014
P7	Notice on the issuance of an action plan to further improve medical services	National Health and Family Planning Commission, National Bureau of Chinese Medicine	12 January 2015
P8	Notice on the issuance of the main points of health and family planning work in 2015	National Health and Family Planning Commission	14 January 2015
P9	Notice on agreeing to carry out pilot telemedicine policy work in five provinces and regions, including Ningxia and Yunnan	General Office of the National Development and Reform Commission, General Office of the National Health and Family Planning Commission	15 January 2015
P10	Notice on the issuance of the outline of the national healthcare service system planning (2015-2020)	Office Council of the State	6 March 2015
P11	Implementation opinions on further strengthening the construction of rural doctors	Office Council of the State	6 March 2015
P12	Implementation opinions on the comprehensive reform of county-level public hospitals	Office Council of the State	23 April 2015
P13	Notice on the issuance of the summary of the work of deepening the reform of the medical and health system in 2014 and the key work tasks in 2015	Office Council of the State	26 April 2015
P14	Opinions on the pilot comprehensive reform of urban public hospitals	Office Council of the State	6 May 2015
P15	Notice on forwarding the national mental health work plan (2015-2020) of the health and family planning commission and other departments	Office Council of the State	4 June 2015
P16	Opinions on the use of big data to enhance services and supervision of market subjects	Office Council of the State	24 June 2015
P17	Guiding opinions of the state council on actively promoting "internet" action	State Council	1 July 2015
P18	Notice on the issuance of the action outline for the promotion of big data development	State Council	31 August 2015
P19	Guidance on promoting the construction of graded treatment system	State Council	8 September 2015
P20	Guidance on further standardizing the management of community health services and improving the quality of services	National Health and Family Planning Commission, State Administration of Traditional Chinese Medicine	17 November 2015
P21	Notice on promoting the integration of medical and health care with elderly services	Office Council of the State	18 November 2015
P22	"Health China 2030" Plan	The Central Committee of the Communist Party of China, the State Council	1 January 2016
P23	Notice on the issuance of the outline of the strategic plan for the development of Chinese medicine (2016- 2030)	State Council	22 February 2016
P24	Guidance on promoting the healthy development of the pharmaceutical industry	Office Council of the State	4 March 2016
P25	Notice on the issuance of key work tasks for deepening the reform of the medical and health system in 2016	Office Council of the State	21 April 2016
P26	Notice on the issuance of opinions on strengthening the reform and development of medical and health services for children	National Health and Family Planning Commission, National Development and Reform Commission, Ministry of Education, etc.	13 May 2016
P27	Guiding principles for implementing health poverty alleviation initiatives	National Health and Family Planning Commission, State Council Poverty Alleviation Office, National Development and Reform Commission, etc.	20 June 2016
P28	Guidance on promoting and regulating the development of health care big data applications	Office Council of the State	21 June 2016
P29	Outline of the national strategy for the development of information technology	General Office of the Central Committee of the Communist Party of China and General Office of the State Council	1 July 2016
P30	Notice on the issuance of the national science and technology innovation plan for the 13th Five-Year Plan	State Council	28 July 2016
P31	Notice on the invitation to organize the declaration of special projects for building innovation capacity in the field of "internet plus"	General Office of the National Development and Reform Commission	26 August 2016
P32	Several opinions on strengthening basic health care services throughout the childbirth process	National Health and Family Planning Commission, National Development and Reform Commission, Ministry of Education, etc.	14 October 2016
P33	Notice on the issuance of management measures for the construction of national demonstration areas for comprehensive prevention and control of chronic diseases (revised 2016)	General Office of the National Health and Family Planning Commission	20 October 2016
P34	Announcement No. 58 of 2016 - announcement of the guidance catalogue for key areas of service exports	Ministry of Commerce	25 October 2016
P35	Notice on the issuance of the "pharmaceutical industry development planning guidelines”	Ministry of Industry and Information Technology, National Development and Reform Commission, Ministry of Science and Technology, etc.	26 October 2016
P36	Notice on the issuance of the 13th Five-Year Plan for poverty alleviation	State Council	23 November 2016
P37	Notice on the issuance of the national strategic emerging industries development plan for the thirteenth Five-Year Plan	State Council	29 November 2016
P38	Notice on issuing the national informatization plan for the 13th Five-Year Plan	State Council	15 December 2016
P39	Notice on the issuance of the 13th Five-Year Plan for health and wellness	State Council	27 December 2016
P40	Notice on the issuance of the 13th Five-Year Plan for deepening the reform of the medical and health system	State Council	27 December 2016
P41	Notice on the issuance of the medium- and long-term plan for the prevention and control of chronic diseases in China (2017-2025)	Office Council the State	22 January 2017
P42	Circular on the issuance of the 13th Five- Year Plan for the prevention and control of tuberculosis	Office Council the State	1 February 2017
P43	Notice on the adjustment of the counterpart relationship of some local tertiary hospitals in helping county hospitals in poverty-stricken counties	General Office of the National Health and Family Planning Commission, General Department of the State Council Poverty Alleviation Office, Office of the State Administration of Traditional Chinese Medicine	19 April 19 2017
P44	Guidance on promoting the construction and development of medical consortia	Office Council the State	23 April 2017
P45	Guiding opinions on promoting the development of health tourism	National Health and Family Planning Commission, National Development and Reform Commission, Ministry of Finance	12 May 2017
P46	Opinions on supporting social forces to provide multi-level and diversified medical services	Office of the State Council	16 May 2017
P47	Notice on the issuance of the development plan for a new generation of artificial intelligence	State Council	8 July 2017
P48	Development plan for national clinical medical research centers (2017- 2021) (attached: management measures for national clinical medical research centers (revised in 2017))	Ministry of Science and Technology, National Health and Family Planning Commission, Department of Logistics and Security of the Military Commission, General Administration of Food and Drug Administration	19 July 2017
P49	Notice on strengthening the management of health education information services	General Office of the National Health and Family Planning Commission	21 August 2017
P50	Notice on the national basic public health service project for 2017	National Health and Family Planning Commission, Ministry of Finance, State Administration of Traditional Chinese Medicine	23 August 2017
P51	Guidance on promoting the integration and development of Chinese medicine health services with the internet	State Administration of Chinese Medicine	4 December 2017
P52	Notice on the issuance of guidelines for the construction and management of critical maternal and neonatal treatment centers	General Office of the National Health and Family Planning Commission	8 December 2017
P53	Notice on the issuance of the action plan for further improvement of medical services (2018-2020)	National Health and Family Planning Commission, State Administration of Traditional Chinese Medicine	29 December 2017
P54	Opinions of the general office of the state council on promoting the development of "internet plus medical health"	Office of the State Council	25 April 2018
P55	Notice on the national basic public health service project for 2018	National Health Care Commission, Ministry of Finance, State Administration of Traditional Chinese Medicine	13 June 2018
P56	Notice on the in-depth development of "Internet plus medical health" activities for the benefit of the people	National Health Care Commission, State Administration of Traditional Chinese Medicine	10 July 2018
P57	Several opinions on strengthening the work of ethnic minority medicine in the new era	State Administration of Traditional Chinese Medicine, State Ethnic Affairs Commission, National Development and Reform Commission, etc.	12 July 2018
P58	Notice on the issuance of national health care big data standards, security and services management measures (for trial implementation)	National Health and Wellness Committee	12 July 2018
P59	Measures for the administration of internet medical treatment (for trial implementation) (with: measures for the administration of internet hospitals (for trial implementation) and the code for the administration of telemedicine services (for trial implementation))	National Health Care Commission, State Administration of Traditional Chinese Medicine	17 July 2018
P60	Notice on the distribution of key tasks of the national television and telephone conference on deepening the reform of "administration and service" and transforming the functions of the government	Office of the State Council	5 August 2018
P61	Notice on the issuance of key tasks for the second half of 2018 for deepening the reform of the medical and health system	Office of the State Council	20 August 2018
P62	Notice on further promoting the construction of information technology in medical institutions with electronic medical records as the core	National Health and Wellness Committee	22 August 2018
P63	Opinions on strengthening collaboration between medicine and education to implement the excellent doctor education and training program 2.0	Ministry of Education, National Health Care Commission, State Administration of Traditional Chinese Medicine	17 September 2018
P64	Notice on the issuance of the implementation plan for improving the institutional mechanism for promoting consumption (2018-2020)	Office of the State Council	24 September 2018
P65	Notice on the issuance of the implementation plan for the three-year plan of action for medical security and poverty alleviation (2018-2020)	National Health Insurance Administration, Ministry of Finance, State Council Poverty Alleviation Office	30 September 2018
P66	Notice on the issuance of guidance for public hospital to conduct online payment business	General Office of the National Health and Wellness Commission	15 October 2018
P67	Opinions on accelerating the high-quality development of pharmacy services	National Health and Wellness Commission, State Administration of Traditional Chinese Medicine	21 November 2018
P68	Opinions on accelerating the popularization and application of electronic health cards	General Office of the National Health and Wellness Commission	13 December 2018
P69	Notice on the pilot project of "internet plus nursing services	General Office of the National Health and Wellness Commission	22 January 2019
P70	Opinions on the division of work among key departments for the implementation of the government work report (2019)	State Council	29 March 2019
P71	Notice on the issuance of key tasks for deepening the reform of the medical and health system in 2019	Office of the State Council	23 May 2019
P72	Opinions on promoting the sustainable, healthy and standardized development of socially run hospitals	National Health and Health Commission, National Development and Reform Commission, Ministry of Science and Technology	10 June 2019
P73	Guidance on improving the pricing and health insurance payment policies for "Internet plus" medical services	National Health Insurance Agency	17 August 2019
P74	Notice on the issuance of the action plan for promoting high-quality development of health industry (2019-2022)	National Development and Reform Commission, Ministry of Education, Ministry of Science and Technology, etc.	28 August 2019
P75	Notice on supporting the promotion of internet poverty alleviation projects	General Office of the National Development and Reform Commission, Secretary Bureau of the Central Internet Information Office, General Office of the Ministry of Agriculture and Rural Affairs, Agricultural Development Bank of China	10 September 2019
P76	Notice on the issuance of the implementation plan to curb the spread of AIDS (2019-2022)	National Health Commission, Central Propaganda Department, Central Committee of Political Science and Law, etc.	11 September 2019
P77	Guidance on the high-quality development of the service industry in the new era	National Development and Reform Commission, General Administration of Market Regulation	2 October 2019
P78	Opinions on further promoting the development of combined medical and health care	National Health Care Commission, Ministry of Civil Affairs, National Development and Reform Commission, etc.	23 October 2019
P79	Opinions on promoting the development of "Internet plus social services	National Development and Reform Commission, Ministry of Education, Ministry of Civil Affairs	6 December 2019
P80	Notice on the work of Internet consultation services in the prevention and control of epidemics	General Office of the National Health and Wellness Commission	6 February 2020
P81	Notice on the issuance of opinions on strengthening the management of pharmacy in medical institutions to promote rational use of drugs	National Health and Wellness Commission, Ministry of Education, Ministry of Finance, etc.	21 February 2020
P82	Notice on the coordinated promotion of health and poverty alleviation during the prevention and control of the new crown pneumonia outbreak	General Office of the National Health and Wellness Commission	24 February 2020
P83	Opinions on deepening the reform of the medical security system	The Central Committee of the Communist Party of China and the State Council	25 February 2020
P84	Notice on the further implementation of scientific prevention and control, precise treatment, zoning and grading requirements to improve the management of medica services during the epidemic	General Office of the National Health and Wellness Commission	26 February 2020
P85	Guidance on promoting "Internet plus" health insurance services during the prevention and control of the new pneumonia epidemic	National Health Insurance Agency, National Health Care Commission	28 February 2020
P86	Notice on the work of primary health care institutions in the prevention and control of the new crown pneumonia epidemic in a categorized and precise manner	General Office of the National Health and Wellness Commission	1 March 2020
P87	Notice on the issuance of the guidelines on the construction and application of information technology for community prevention and control of the new coronary pneumonia epidemic	General Office of the Ministry of Civil Affairs, Secretary Bureau of the Central Internet Information Office, General Office of the Ministry of Industry and Information Technology, General Office of the National Health and Wellness Commission	2 March 2020
P88	Notice on the implementation plan for promoting the action of "using the cloud to empower wisdom" to foster the development of new economy	National Development and Reform Commission, Central Internet Information Office	7 April 2020
P89	Notice on the technical specifications and financial management of the "Internet plus medical services" project in public medica institutions	National Health and Wellness Commission, State Administration of Traditional Chinese Medicine	8 May 2020
P90	Notice on further improving the appointment and treatment system and strengthening the construction of smart hospitals	General Office of the National Health and Wellness Commission	21 May 2020
P91	Notice on the basic public health service project in 2020	National Health and Health Commission, Ministry of Finance, State Administration of Traditional Chinese Medicine	12 June 2020
P92	Notice on better information technology to support regular epidemic prevention and control work	General Office of the National Health and Wellness Commission	28 June 2020
P93	Notice on the issuance of measures for the management of medical consortia (for trial implementation)	Health and Wellness Commission, Chinese Medicine Bureau	9 July 2020
P94	Opinions on supporting the healthy development of new industries and new models to activate the consumer market and drive the expansion of employment	National Development and Reform Commission, Central Internet Information Office, Ministry of Industry and Information Technology, etc.	14 July 2020
P95	Implementation on further optimizing the business environment to better serve market players	Office of the State Council	15 July 2020
P96	Notice on the issuance of key tasks for the second half of 2020 to deepen the reform of the medical and health system	Office of the State Council	16 July 2020
P97	Opinions on accelerating the development of new types of consumption led by new industries and new modes	Office of the State Council	16 September 2020
P98	Notice on the issuance of guidelines for the management of medical and health care institutions (for trial implementation)	General Office of the National Health and Health Commission, General Office of the Ministry of Civil Affairs, Office of the State Administration of Traditional Chinese Medicine	27 September 2020
P99	Opinions on strengthening the construction of a standardized system of health information for all	General Office of the National Health and Wellness Commission and Office of the State Administration of Traditional Chinese Medicine	27 September 2020
P100	Notice on further strengthening the capacity building of telemedicine networks	General Office of the Ministry of Industry and Information Technology and General Office of the National Health and Wellness Commission	22 October 2020
P101	Guidance on actively promoting the payment of medical insurance for "Internet plus" medical services	Medical Insurance Board	24 October 2020
P102	Notice on deepening the reform of "management and service" and optimizing the business environment (2020)	Office of the State Council	1 November 2020
P103	Notice on the "Five-One" service initiative for the further promotion of "internet plus medical health"	National Health Care Commission, National Health Insurance Administration, National Administration of Traditional Chinese Medicine	4 December 2020
P104	Notice on the issuance of service guidelines for contracted cooperation between medical and health institutions and elderly service organizations (for trial implementation)	General Office of the National Health and Health Commission, General Office of the Ministry of Civil Affairs, Office of the State Administration of Traditional Chinese Medicine	11 December 2020
P105	Notice on strengthening home medical services for the elderly	General Office of the National Health and Wellness Commission, Office of the State Administration of Traditional Chinese Medicine	17 December 2020
P106	Notice on the issuance of guidance on further regulating medical practices to promote reasonable medical examinations	National Health Care Commission, National Development and Reform Commission, Ministry of Finance, etc.	29 December 2020
P107	Notice on the issuance of internal control management measures for public hospitals	National Health and Wellness Commission, State Administration of Traditional Chinese Medicine	1 January 2021
P108	Notice on the issuance of the implementation opinions on consolidating and expanding the achievements of poverty alleviation through health and effectively linking with rural revitalization	National Health Commission, National Development and Reform Commission, Ministry of Industry and Information Technology, etc.	1 February 2021
P109	Notice on the issuance of the implementation plan for accelerating the cultivation of new types of consumption	Development and Reform Commission, Central Internet Information Office, Ministry of Education	22 March 2021
P110	Opinions on promoting the high-quality development of public hospitals	Office of the State Council	14 May 2021
P111	Notice on the issuance of opinions on accelerating the development of rehabilitation medical work	Health and Welfare Commission, Development and Reform Commission, Ministry of Education, etc.	8 June 2021
P112	Notice on the issuance of the "5G Application" sailing action plan (2021- 2023)	Ministry of Industry and Information Technology, Office of the Central Committee for Network Security and Informatization, National Development and Reform Commission, etc.	5 July 2021
P113	Opinions on optimizing user-friendly services in the field of health insurance	National Health Insurance Agency	16 July 2021
P114	Notice on the issuance of the program for the development of Chinese women and the program for the development of Chinese children (2021)	State Council	8 September 2021
P115	Notice on the issuance of the action to promote high- quality development of public hospitals (2021-2025)	National Health and Wellness Commission, State Administration of Traditional Chinese Medicine	14 September 2021
P116	Circular on the issuance of the 14th Five- Year Plan for universal medical coverage	Office of the State Council	23 September 2021
P117	Notice on the issuance of the "14th Five-Year Plan" for the development of big data industry	Ministry of Industry and Information Technology	15 November 2021
P118	Notice on the issuance of the 14th Five-Year Plan for the Development of the Digital Economy	State Council	12 December 2021
P119	Guidance on medical insurance support for the development of Chinese medicine inheritance and innovation	National Health Insurance Administration, State Administration of Traditional Chinese Medicine	14 December 2021
P120	Notice on the issuance of a work plan for the 14th Five-Year Plan period for tertiary hospitals to help county-level hospitals	National Health and Wellness Commission, National Bureau of Rural Development, National Bureau of Chinese Medicine	16 December 2021
P121	Notice on the identification of the second batch of pilot organizations for combined medical and health care tele-coordination services for the elderly	General Office of the National Health and Wellness Commission	29 December 2021
P122	Notice on the issuance of the national eye health plan for the 14th Five-Year Plan (2021-2025)	National Health and Wellness Commission	4 January 2022
P123	Notice on the issuance of the "14th Five-Year Plan" for health and wellness standardization work	National Health and Wellness Commission	11 January 2022
P124	Opinions on certain special measures to ease market access in Shenzhen’s demonstration zone for the construction of socialism with Chinese characteristics	National Development and Reform Commission, Ministry of Commerce	24 January 2022
P125	Notice on strengthening information technology to support the prevention and control of pneumonia outbreaks caused by novel coronavirus infections	General Office of the National Health and Wellness Commission	3 February 2022
P126	Notice on the issuance of the rules for the regulation of internet medical treatment (for trial implementation)	General Office of the National Health and Wellness Commission and Office of the State Administration of Traditional Chinese Medicine	8 February 2022
P127	Notice on regulating the management of branch hospital areas in public hospitals	National Health and Wellness Commission	24 February 2022
P128	Notice on the issuance of the "14th Five-Year Plan" action plan for the basic Chinese medicine service capacity enhancement project	State Administration of Traditional Chinese Medicine, National Health and Wellness Commission, National Development and Reform Commission, etc.	8 March 2022
P129	Notice on the issuance of the national health plan for the 14th Five-Year Plan	Office of the State Council	27 April 2022
P130	Notice on deepening the reform of the medical and health system	Office of the State Council	4 May 2022
P131	Guidance on accelerating scene innovation to promote high quality economic development with high level application of artificial intelligence	Ministry of Science and Technology and six other departments	29 July 2022
P132	Guidance on accelerating the development of virtual reality industry	Ministry of Industry and Information Technology	8 August 2022
P133	Notice on the issuance of cyber security management measures for medical and health institutions	National Health and Wellness Commission, National Bureau of Chinese Medicine, National Bureau of Disease Control and Prevention	8 August 2022
P134	Notice on the issuance of the 14th Five-Year Plan for health informatization for all people	National Health Commission, State Administration of Traditional Chinese Medicine, National Bureau of Disease Control and Prevention	7 November 2022

## Appendix B

**Table A2:** Evaluation sample of internet healthcare policies.

NO.	Policy Document	Issuing Department	Issuing Date
P1	Notice on conducting policy pilot work for remote medical care in nursing homes	National Development and Reform Commission, Ministry of Civil Affairs, National Health and Family Planning Commission	16 June 2014
P2	Opinion on promoting remote medical services in medical institutions	National Health and Family Planning Commission	21 August 2014
P3	Notice on approving policy pilot work for remote medical care in five provinces and regions, including Ningxia and Yunnan	National Development and Reform Commission Office, National Health and Family Planning Commission Office	15 January 2015
P4	Guiding opinions on promoting and regulating the development of health and medical big data applications	General Office of the State Council	21 June 2016
P5	Guiding opinions on promoting the integration and development of traditional Chinese medicine health services with the internet	National Administration of Traditional Chinese Medicine	4 December 2017
P6	Opinion on promoting the development of “Internet Plus Medical and Health”.	General Office of the State Council	25 April 2018
P7	Notice on carrying out activities for the convenience and benefit of the people in the “Internet Plus Medical and Health” field	National Health Commission, State Administration of Traditional Chinese Medicine	10 July 2018
P8	Notice on issuing the National Health and Medical Big Data Standards, Security, and Service Management Measures (Trial)	National Health Commission	12 July 2018
P9	Notice on further promoting the information construction of medical institutions with electronic medical records as the core	National Health Commission	22 August 2018
P10	Notice on issuing guiding opinions for public hospitals to carry out online payment business	National Health Commission Office	15 October 2018
P11	Notice on conducting pilot work for “Internet Plus Nursing Services”.	National Health Commission Office	22 January 2019
P12	Guiding opinions on improving the pricing and medical insurance payment policies for “Internet Plus” medical services	National Healthcare Security Administration	17 August 2019
P13	Notice further improving the appointment and diagnosis system and strengthening the construction of smart hospitals	National Health Commission Office	21 May 2020
P14	Notice on further strengthening the network capacity building for remote medical care	Ministry of Industry and Information Technology Office, National Health Commission Office	22 October 2020
P15	Guiding opinions on actively promoting the medical insurance payment work for “Internet Plus” medical services	National Healthcare Security Administration	24 October 2020
P16	Notice on further promoting the “Five Ones” service action in the “Internet Plus Medical and Health” field	National Health Commission, National Healthcare Security Administration, State Administration of Traditional Chinese Medicine	4 December 2020
